# Novel Conjugated Polymers Prepared by Direct (Hetero) arylation: An Eco-Friendly Tool for Organic Electronics

**DOI:** 10.3390/molecules23020408

**Published:** 2018-02-13

**Authors:** Fuchuan Liu, Yangqian Zhang, Hang Wang, Shiming Zhang

**Affiliations:** 1 Key Laboratory of Flexible Electronics (KLOFE) & Institute of Advanced Materials (IAM), Jiangsu National Synergetic Innovation Center for Advanced Materials (SICAM), Nanjing Tech University (Nanjing Tech), 30 South Puzhu Road, Nanjing 211816, China; fcliu@njtech.edu.cn (F.L.); iamyqzhang@njtech.edu.cn (Y.Z.); iamwanghang@njtech.edu.cn (H.W.); 2 Nanjing Kuo Hua Electronics Technology Pte. Ltd., Innovation Building B816, Xinmofan Road 5, Nanjing 210009, China

**Keywords:** direct (hetero)arylation polymerization, phthalimide, thiocarbonyl, conjugated polymer, organic electronics

## Abstract

The phthalimide (PhI) moiety has been attracting more attention as an excellent acceptor building block in donor-acceptor (D-A) conjugated polymers. In this paper; three D-A conjugated polymers with or without thiocarbonyl moieties are successfully prepared by the direct (hetero)-arylation polymerization (DHAP), which is an atom efficient and facile synthetic strategy to obtain polymer materials. Compared with the traditional carbon-carbon coupling reactions, this method possesses more advantages, including: fewer synthetic steps, avoidance of the preparation of the organometallic reagents, higher atom economy and fewer toxic byproducts, better compatibility with chemically sensitive functional groups and so on. All three of these designed PhI-based polymers exhibited favourable optoelectronic and thermal performance. The optical, thermodynamic and electrochemical properties of the synthesized polymers were systematically investigated using ultraviolet-visible (UV-vis) spectroscopy, thermogravimetric analysis (TGA), differential scanning calorimetry (DSC) and cyclic voltammetry (CV). The results of these three polymers indicated that thionation of the carbonyl was a highly effective methods to improve the properties of PhI-based polymers; and provided impetus for the development of thionated PhI derivatives for organic electronic applications.

## 1. Introduction 

Because of the low cost, light weight, flexibility property, π-conjugated polymers have been widely utilized for organic semiconductor devices, including organic light-emitting diodes (OLEDs) [1,2], organic field-effect transistors (OFETs) [3,4,5] and organic solar cells (OSCs) [6,7,8]. In recent years, π-conjugated polymers based on the donor (D) and acceptor (A) alternative structures are of great importance and interest on account of their special optical, thermal and electrochemical properties and potential applications in organic semiconductor devices. Imide-based molecules are broadly used as electron deficient materials due to their high photochemical stability, ease of synthetic modification, and outstanding charge transmission ability [9,10,11]. Many of the best-performing materials for OLEDs, OFETs and OSCs contain an electron-withdrawing carbonyl group in the form of an amide or imide functionality, such as diketopyrrolopyrrole (DPP) [12,13], thieno[3,4-*c*]pyrrole-4,6-dione (TPD) [14,15], perylene diimide (PDI) [9,16], naphthalene diimide (NDI) [17,18] and so on [19,20,21]. The strong electron-withdrawing nature of imides also makes them excellent candidates as building blocks for photoactive materials. To date, a majority of materials use such moieties in the core of the small molecules [22,23] or as acceptor units in D-A conjugated polymers [11,24].

In the last five years, many reports have detailed the use of direct (hetero)arylation polymerization (DHAP) [25,26,27,28,29], which is a novel and powerful method for the synthesis of π-conjugated small molecules and polymers with a large variety of small molecule building blocks. Compared with the traditional metal-catalyzed cross-coupling such as the Stille coupling and Suzuki coupling, DHAP allows the formation of carbon-carbon bonds between arenes and aryl halides directly and possesses many advantages such as the needless of prefunctionalization of monomers using hazardous reagents; reducing the reaction steps and cost; avoiding the employing of dangerous reagents as well as the formation of toxic byproducts, and decreasing environmental pollution [30,31,32]. So far, there were a lot of articles and reviews reported about DHAP [32,33], which could really give the reader essential information about the DHAP reaction and the advances contained in the paper with respect to state-of-the-art. And synthesizing organic semiconductor materials also included many different electron deficiency units such as DPP, TPD and so on. Punzi and coworkers successfully synthesized TEG-substituted DPP-based low band gap polymers via the DHAP method firstly [34]. The same group also applied this synthetic methodology to the TPD unit and the reaction was successfully carried out in green solvents [35], which really corresponded to an “environmentally benign” procedure, so the DHAP synthetic methodology could provide a green, convenient, and inexpensive shortcut to more complex materials, including small molecules and polymers for organic electronics, even for production on an industrial scale [36].

Designs principle for new π-conjugated polymers for organic electronic applications include lowering the LUMO energy level for higher stability and improving the intermolecular and intramolecular interactions for efficient charge transport [37,38]. A far less studied electron deficient functional group is the thiocarbonyl group, where that is carbonyl oxygen is replaced with sulfur [39,40]. Previously, this functionality had been mainly studied in biological chemistry [41,42]. However, the introduction of thionating materials for organic electronics has been gradually introduced [43,44,45,46]. These thionation methods typically lead to a lowering of the LUMO level and increaed electron affinity. Furthermore, thionation gives rise to the possibility of S–S contacts, which can increase the intermolecular and intramolecular interactions to obtain high charge mobility.

Recent studies have demonstrated that the tailoring of molecular structures of π-conjugated polymers may result in new materials with unusual and exceptional properties. The LUMO levels of imide compounds can be stabilized by introducing strong electron-withdrawing groups into the molecules’ framework [38,47,48]. The simple thionation of imide compounds not only increases the intermolecular interactions, but also reduces their LUMO levels compared with those of their parent compounds [49,50,51,52]. Moreover, the electron mobility of thionated compounds is higher than those of parent compounds under a nitrogen atmosphere. The LUMO levels of thionated molecules were much lower, indicating the electron-withdrawing ability of the thioimide group is stronger than the imide group. Although the electronegativity of oxygen is stronger than that of sulfur, oxygen atoms are comparatively small and their electronic orbitals are very tight. The interelectronic repulsion induced by an additional electron is higher in oxygen than that in sulfur. Therefore, the electron-withdrawing ability of the imide group is lower than expected [45]. The low lying LUMO level could improve the air-stability of the devices and on account of the intense S–S intermolecule and intramolecule interactions, the material could achieve higher charge mobility.

To date, several studies have illustrated the importance of thioimides in improving the properties of these small molecules by lowering the LUMO energy level and increasing the charge mobility even further [45,46,50]. The stabilization of the LUMO level can improve the air-stability of the devices. Furthermore, the intense S–S contact increases the intermolecular electronic coupling in the solid state, thereby enhancing the charge mobility of the device. Therefore, thionation not only increases the electron mobility of the original imide molecules but also improves the air-stability of these compounds.

Phthalimide (PhI) is a famous building block for constructing conjugated polymers used in organic electronic devices [53,54]. Over the past several years, the PhI unit has emerged as one of the most widely used acceptor units to construct high performance materials for organic semiconductor applications [55,56,57]. The PhI molecule is electron-withdrawing, on account of the electrophilic imide group, which plays an important role in D-A conjugated polymers. It possesses a small aromatic core which is favorable for tuning its chemical structure and physical properties. Moreover, the original source of PhI, phthalic anhydride, is much cheaper and easier to prepare, which provides an advantage for large scale synthesis. More importantly, incorporation of PhI units between linking bridges could also increase the intermolecular and intramolecular interactions which would be beneficial for the charge transport. Bithiophene (BTh) has been widely employed as the donor unit for high mobility polymers in organic electronic devices due to its high degree of polymer backbone planarity and the ordering film morphology [56,57].

Because of the promising potential of PhI-based conjugated polymers as practical organic electronic materials, efficient DHAP of PhI-based monomers are highly desirable. As we all know, the carbon-hydrogen bonds on the benzene ring possess lower reaction activity, so it is lightly difficult for them to be involved in the reaction [58,59,60]. DHAP is a technology to develop sustainable, atom-efficient, and environmentally benign polymerization method for the large-scale synthesis of high performance copolymers with less branching defects, allowing the formation of carbon-carbon bonds between carbon-hydrogen bonds and carbon-halogen bonds directly. Thus, it is necessary to enlarge the scope of application of DHAP with the carbon-hydrogen bones based on any aromatic rings participating in this kind of reaction. In our study, we have systematically investigated the reaction conditions of DHAP for PhI units. The results from this study provide a generally applicable methodology for the broad application of DHAP to synthesize more useful π-conjugated materials.

Among the well-established chemical modification strategies for conjugated polymers, substitution of the imide oxygen atoms with sulfur, known as thionation, which renders the core more electron deficient, may provide an excellent approach to tuning the optoelectronic properties of materials [46,50,61]. This study aims to investigate the effect of thionation of imide groups on the conjugated polymers. In contrast to the original imide, the thionation of PhI-based polymer has been barely studied, and fewer considering the limited understanding of the difference between the original and thionated polymers, studies addressing the design and synthesis of thionated material should be of great interest.

In this work, we described the synthesis of a series of novel polymers with or without thiocarbonyl by optimal DHAP method and characterization of the optical, electrochemical and thermal properties. We anticipated that by replacing one or two of the carbonyl groups with thiocarbonyl, we could tune the optoelectronic properties in this system and prepare materials with broad absorption region and low LUMO energy level, which is attractive for potential application in organic semiconductor devices.

## 2. Results and Discussion

### 2.1. Synthesis of the Polymers

**Scheme 1:**
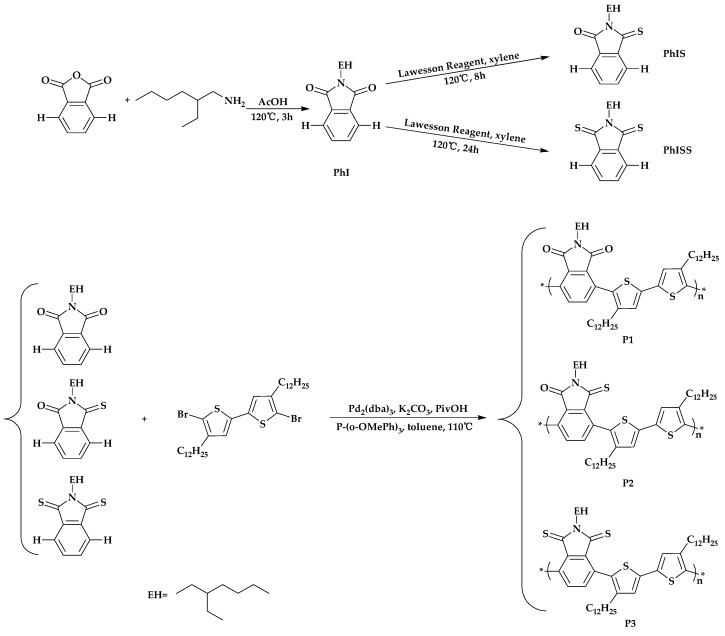
Synthetic route to the polymers P1, P2 and P3.

The synthetic routes for monomers and polymers are shown in Scheme 1. The compound PhI was easily synthesized from phthalic anhydride and 2-ethylhexylamine. Then the compounds PhIS and PhISS were synthesized by the thionation of the PhI under different reaction conditions. The polymers with electron-rich bithiophene as donor and electron-deficient PhI, PhIS or PhISS as acceptor units were synthesized by DHAP with 1:1 monomer ratio in the presence of tris(dibenzylideneacetone)dipalladium (Pd_2_(dba)_3_) catalyst (5 mol %), phosphine ligand (P-(o-OMePh)_3_) (10 mol %), potassium carbonate (K_2_CO_3_) (2.5 equiv.) and pivalic acid (PivOH) (30 mol %) at 110 °C for 72 h to give polymer materials in moderate yields. The crude polymers were purified by precipitating in methanol and washing sequentially with methanol and hexane in a Soxhlet extractor and the residue was extracted with hot chloroform in an extractor, respectively. After removal of solvent, polymer solid materials were collected.

The chemical structure of the polymers was verified by nuclear magnetic resonance (NMR) spectroscopy. The polymers showed good solubility in common organic solvents such as chloroform (CF), toluene (Tol), chlorobenzene (CB), and dichlorobenzene (DCB) on account of the multiple linear and branched side chains. The number-average molecular weight (M_n_), weight-average molecular weight (Mw) and polydispersity index (PDI) of the polymers were determined by gel permeation chromatography (GPC) with dichlorobenzene (DCB) as eluent against polystyrene standards.

### 2.2. Computational Analysis

**Figure 1:**
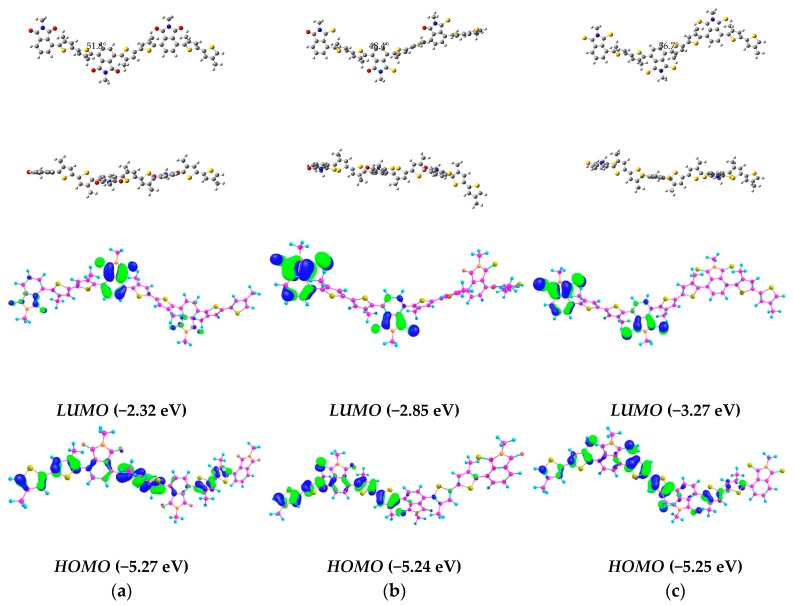
Optimized molecular geometry and the frontier orbital density distributions for P1 (**a**); P2 (**b**) and P3 (**c**) via DFT calculations (B3LYP/6-31G (d, p)). Dihedral angels between adjacent rings (degrees, °) were shown in black text (top structures). HOMO and LUMO molecular orbital diagrams were shown below with calculated energy levels.

To support our assumption that the thionated acceptor units should decrease the LUMO energy level of the PhI-based polymers, we utilized gas-phase density functional theory (DFT) to analyze truncated structures and compared to the parent PhI-based polymer. All DFT calculations were performed with the Gaussian 09 and the molecular geometries for the three polymers were optimized at the B3LYP level of theory with the 6-31G (d, p) basis set. To simplify the calculation, all the alkyl groups were replaced with methyl groups. Figure 1 shows the calculated molecular orbital geometry and energy levels on the model compound of the polymers. For the bithiophene structure, the PhI and thionated PhI core are nearly planar. Surprisingly, the backbones of these three polymers are distorted, and the dihedral angels of P1, P2 and P3 between the benzene ring of PhI and the neighboring thiophene ring of bithiophene is about 51.8°, 48.4° and 56.7°, respectively. The larger dihedral angels might be attributed to the steric hindrance of the alkyl group.

The calculated HOMO and LUMO energy level for these two thionated polymers were found to be much deeper than that of parent polymer, supporting the concept that the electron-withdrawing ability of thionated carbonyl is stronger than carbonyl. The HOMO levels of polymers are mainly delocalized over the acceptor units and bithiophene units, therefore, the three polymers have similar deep HOMO levels. In contrast to the HOMO levels, the LUMO levels are distributed on the acceptor units. Since the thionated PhI units show stronger electron-deficient than that of parent PhI unit, polymers P2 and P3 have much lower LUMO levels compared to that of P1, which indicates that the LUMO levels can be effectively lowered by changing the acceptor unit. In addition, when compared to the parent polymer, the HOMO and LUMO energy levels of these thionated polymers are more localized and delocalized, respectively, which again highlights the more electron-withdrawing nature of the thionated carbonyl.

### 2.3. Optical Properties of the Polymers

**Figure 2:**
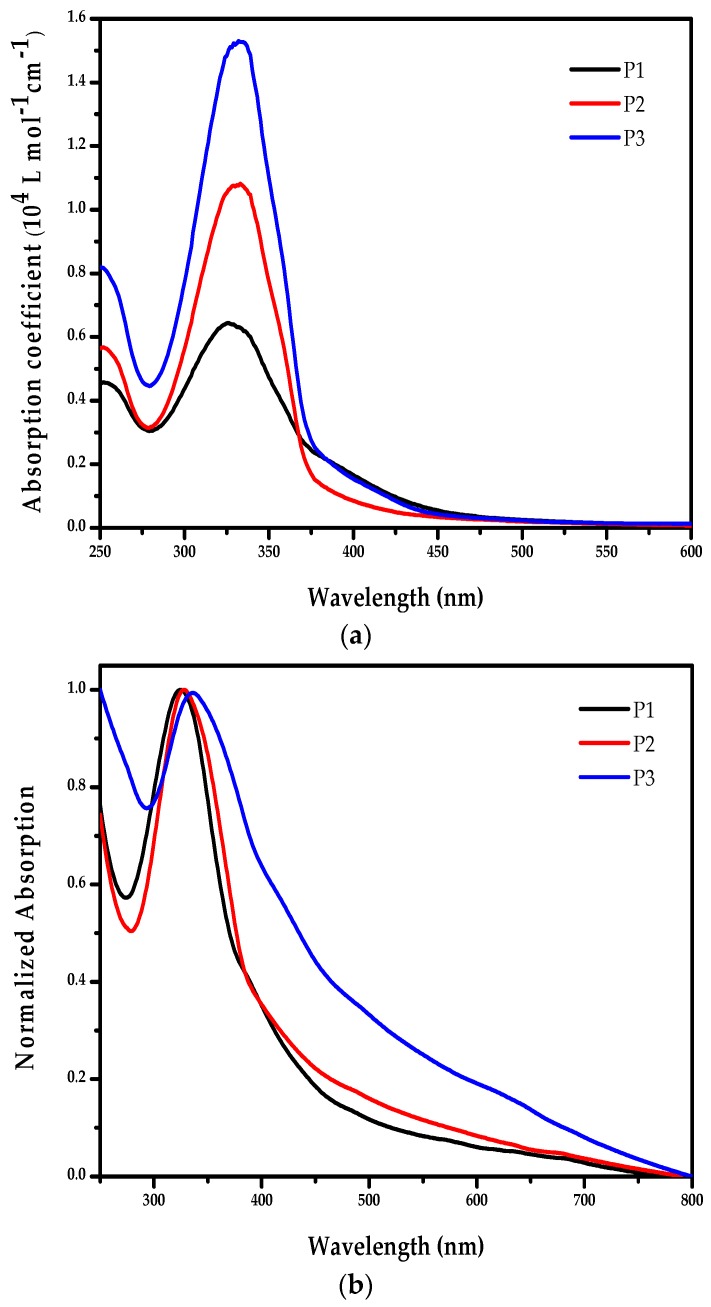
UV-Vis absorption spectra of P1, P2 and P3 in chloroform solution (10^−5^ mol L^−1^) (**a**) and thin film (**b**).

The UV-Vis absorption spectra of P1, P2 and P3 were recorded in dilute chloroform solutions and spin-coated thin films, respectively. The spectra are shown in Figure 2, and the relevant data are summarized in Table 1. Broad absorption bands in the visible region were obtained for both solution and thin film of P1, P2 and P3. The polymers on spin-coated thin films showed broad absorption region from 300 to 800 nm and displayed slightly red-shifted compared with their solution absorption spectrum, which indicated that some interchain interactions occurred in the solid state of the polymers. Compared with the parent compound PhI, the absorption region of the polymers based on the thionated compounds PhIS and PhISS showed an obvious red shift. This improvement of absorption characteristic for polymers might originate from the stronger electron-withdrawing ability of thiocarbonyl and the powerful intermolecular and intramolecular interactions. From the UV-Vis spectrum, we could know the origin of absorption bands in Figure 2a were 364 nm, 378 nm and 383 nm, and in Figure 2b were 459 nm, 468 nm and 484 nm of P1, P2 and P3, respectively. The optical bandgap of polymers was calculated from the absorption edge of thin-films and summarized in Table 1. The results demonstrated that thionated polymers displayed narrower optical bandgap and stronger absorption than the parent compound.

### 2.4. Electrochemical Properties of the Polymers

**Figure 3:**
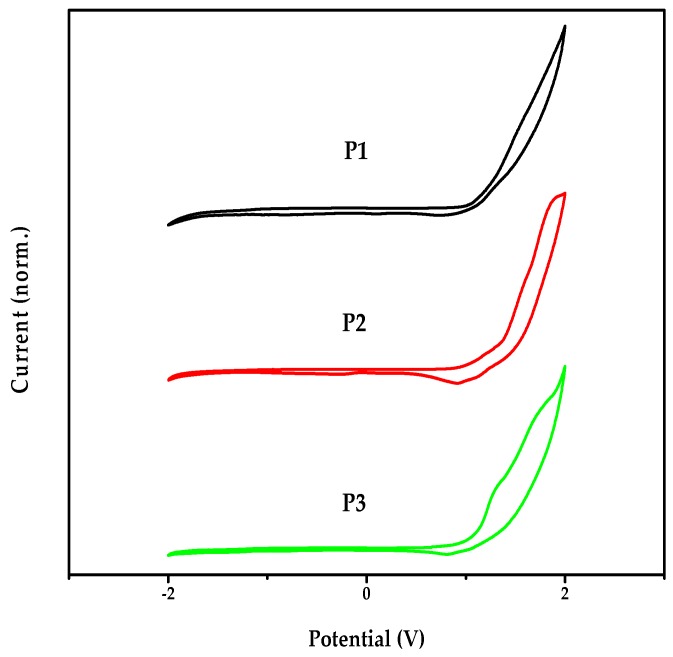
Cyclic voltammogram of P1, P2 and P3.

The highest occupied molecular orbital (HOMO) and lowest unoccupied molecular orbital (LUMO) energy levels of polymers were important parameters which affected the performances of organic semiconductor devices. The electrochemical properties of P1, P2 and P3 were investigated by cyclic voltammetry (CV) where the polymers were dropped as films on the working electrode, respectively. The result showed that all these three polymers showed a partially reversible oxidation wave and no obvious reduction waves were detected, suggesting that they possessed stronger electron donating capacity and were intrinsic p-type semiconductors. Moreover, From the CV results of P1, P2 and P3, the polymers possessed relatively higher oxidation potential that meant lower HOMO energy levels and large energy band gaps, which were beneficial to prevent oxidation reactions under ambient conditions, the much lower HOMO and LUMO energy levels were in an ideal range to ensure good air stability of the devices. Electrochemical measurement was conducted in anhydrous CH_3_CN with 0.1 M Bu_4_NPF_6_ as the supporting electrolyte, a platinum carbon electrode, a platinum wire, and an Ag/AgCl electrode used as the working electrode, the counter electrode and the reference electrode, respectively, with a scan rate at 100 mV s^−1^. The CV curves were calibrated by the ferrocene-ferrocenium (Fc/Fc^+^) redox couple (4.80 eV below the vacuum level). The HOMO energy levels of polymers were calculated from the formula *E_HOMO_* = −(4.80 + $E_{onset}^{ox}$) eV, where the onset oxidation potentials ($E_{onset}^{ox}$) were estimated from the cyclic voltammogram and the corresponding LUMO energy levels were calculated from the equation *E_LUMO_* = *E_HOMO_* + $E_{g}^{opt}$. The CV curves were showed in Figure 3 and the electrochemical data were summarized in Table 1.

### 2.5. Thermal Properties of the Polymers

**Figure 4:**
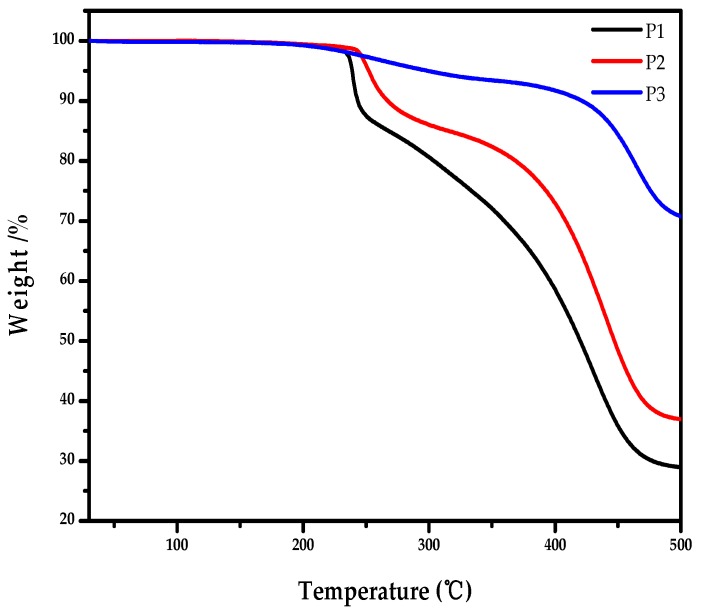
TGA curves for P1, P2 and P3.

**Figure 5:**
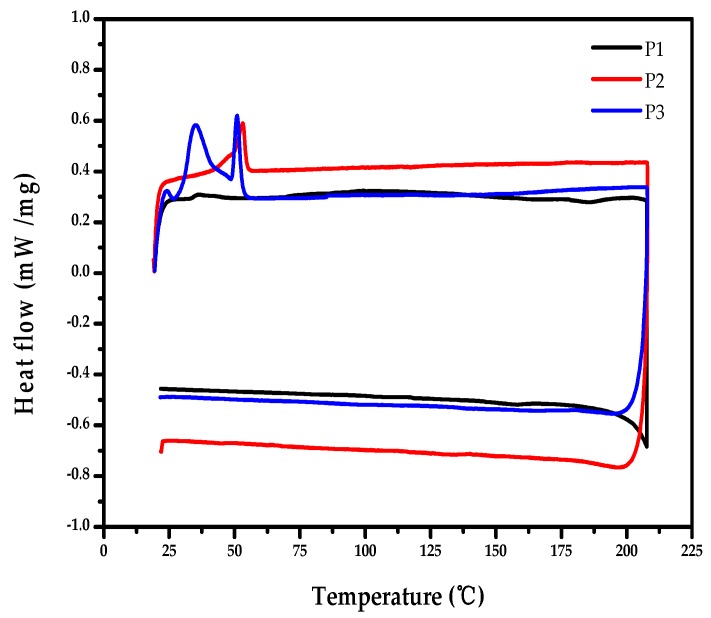
DSC curves for P1, P2 and P3.

Thermal stability is also one of the important factors for the practical application of organic electronic materials. The thermal properties of P1, P2 and P3 were measured by thermogravimetric analysis (TGA) in N_2_ flow with a heating rate of 10 °C min^−1^. The decomposition temperature (T_d_, corresponding to a 5% weight loss) locates at 239 °C, 256 °C and 298 °C for P1, P2 and P3, respectively, indicating that these polymers possessed moderate thermal stability for the application of organic semiconductor devices. From the TGA result, we could know the decomposition temperature (T_d_, corresponding to a 5% weight loss) of P3 was higher than P2 and P1, and P2 was also higher than P1, which was attributed to the introduction of sulfur atom with different number. With introducing sulfur atom with different number, the stronger S–S interaction might be in favour of increasing the T_d_ of polymers, thus P3 exhibited good thermal stability compared to P1 and P2. Phase transition temperatures and enthalpies of P1, P2 and P3 were investigated using differential scanning calorimetry (DSC) in a N_2_ environment with a scanning rate of 10 °C min^−1^. From the DSC results, for all the polymers, there was not obvious endothermic phenomenon occurred in the heating process. However, compared with the parent compound, the polymers based on the thionated monomers showed distinctly liquid crystal transition phenomenon in the cooling process, which was favorable for fabricating high performance organic semiconductor devices. The thermal curves were showed in Figure 4 and Figure 5. The thermal performance data were summarized in Table 1.

## 3. Experimental Section

### 3.1. Materials and Methods

All reagents and starting materials were purchased from commercial sources and used without further purification. Anhydrous solvents were distilled from sodium/benzophenone immediately prior to use.

### 3.2. General Characterization Methods

All NMR spectra were recorded on an AMX300 spectrometer (Bruker, Billerica, MA, USA) at 300 MHz and ^1^H-NMR and ^13^C-NMR spectra were recorded in CDCl_3_ at 303 K. Chemical shifts were reported in δ scale downfield from the peak for tetramethylsilane (TMS). All chemical shifts are quoted in ppm, using the residual solvent peak as a reference standard (CDCl_3_, 7.26 ppm). Mass spectra were measured using a FF-ICR-MS Analyzer (Solarix) in the MALDI mode. The elemental analysis results were determined using a VarioEL CHNS instrument (Elementar, Hesse, Germany). Gel permeation chromatography (GPC) was conducted on a PL-GPC 220 instrument with dichlorobenzene (DCB) as eluent against polystyrene standards. UV-vis absorption spectra were recorded on a UV-1750 spectrophotometer (Shimadzu, city, state abbrev if USA, country) in high performance liquid chromatography (HPLC) grade solvents or quartz plates. Cyclic voltammetry (CV) was carried out on a 660E electrochemical analyzer with a three-electrode cell in a solution of 0.1 M tetrabutylammonium hexafluorophosphate (Bu_4_NPF_6_) as the electrolyte and dissolved in anhydrous CH_3_CN at a scan rate of 100 mV s^−1^. A platinum carbon electrode, a platinum wire, and an Ag/AgCl electrode were used as the working electrode, the counter electrode and the reference electrode, respectively. The potential was scanned from −2.0 to 2.0 V and was calibrated against the ferrocene/ferrocenium (Fc/Fc^+^) internal reference. Thermogravimetric analysis (TGA) was carried out on a TGA2 instrument at a heating rate of 10 °C min^−1^ under N_2_ flow, and differential scanning calorimetry (DSC) was performed on a DSC 214 Polyma instrument at a heating/cooling rate of 10 °C min^−1^ under N_2_ environment.

### 3.3. Synthesis of the Monomers

#### 3.3.1. *N*-(2-Ethylhexyl) Phthalimide (PhI)

In a 250 mL three neck bottom flask, phthalic anhydride (3.0477 g, 20.57 mmol) was dissolved in acetic acid (AcOH, 100 mL) and 2-ethylhexylamine (4.1541 g, 32.14 mmol) was added under nitrogen protection to produce a colorless solution. Then the mixture was heated to 120 °C for 3 h and the color turned light yellow. Next the solvent was removed by rotary evaporation and the residue was dissolved in dichloromethane (DCM, 50 mL) and washed three times with water (100 mL). The organic layer was dried over anhydrous MgSO_4_, filtered and concentrated by rotary evaporation and gained crude yellow oil product. Then the resulting crude yellow oil product was purified via column chromatograph (silica gel, DCM:PE (petroleum ether) = 1:1), giving the final product as a white solid product (4.4316 g, 81.7%). ^1^H-NMR (CDCl_3_) δ = 7.84 (dd, 2H), 7.70 (dd, 2H), 3.58 (d, 2H), 1.83 (m, 1H), 1.31 (m, 8H), 0.92 (t, 6H); ^13^C-NMR (CDCl_3_) δ = 168.65, 133.76, 132.12, 123.09, 41.87, 38.31, 30.53, 28.49, 23.85, 22.94, 13.98, 10.38; *m*/*z* (ESI) 259.1942 (M^+^); Elemental analysis calculated (%): C, 74.10; H, 8.16; N, 5.40. Found (%): C, 73.91; H, 8.32; N, 5.24.

#### 3.3.2. *N*-(2-Ethylhexyl)-thione Phthalimide (PhIS)

*N*-(2-Ethylhexyl) phthalimide (1.6533 g, 6.37 mmol) and Lawesson’s reagent (1.2136 g, 3.01 mmol) were added into a 150 mL three neck bottom flask with xylene (50 mL) under nitrogen protection. Then the mixture was heated overnight to 120 °C in a dark environment. Stirring was stopped and the reaction mixture was cooled down to room temperature to give a deep brown mixture, which was filtered and washed with DCM, giving a reddish solution, and concentrated under reduced pressure, to give a deep reddish crude oily product. This crude deep reddish oil was purified by column chromatography on silica gel (eluent, DCM:PE = 1:2), to afford a rose red oil (1.0825 g, 61.8%). ^1^H-NMR (CDCl_3_) δ = 7.92 (d, 1H), 7.76 (d, 1H), 7.67 (dd, 2H), 3.94 (d, 2H), 2.03 (m, 1H), 1.38–1.25 (m, 8H), 0.94–0.85 (t, 6H); ^13^C-NMR (CDCl_3_) δ = 197.61, 170.37, 137.29, 134.09, 133.15, 127.39, 123.92, 122.81, 45.19, 38.02, 30.81, 28.68, 24.18, 23.18, 14.21, 10.72; *m*/*z* (ESI) 275.2770 (M^+^); Elemental analysis calculated (%): C, 69.78; H, 7.69; N, 5.09. Found (%): C, 69.31; H, 7.90; N, 5.33.

#### 3.3.3. *N*-(2-Ethylhexyl)-dithione Phthalimide (PhISS)

*N*-(2-Ethylhexyl) phthalimide (0.5441 g, 2.11 mmol) and Lawesson’s reagent (3.6096 g, 8.92 mmol) were added into a 150 mL three neck bottom flask with xylene (50 mL) under a nitrogen protection. Then the mixture was heated for 24 h to 120 °C in a dark environment. Stirring was stopped and the mixture was cooled down to room temperature, giving a dark brown mixture, which was filtered and washed with DCM, to afford a deep reddish solution that was concentrated under reduced pressure, giving a dark reddish oil crude product, which was purified by column chromatography on silica gel (eluent, PE), to give a deep red oil (0.5013 g, 80.6%). ^1^H-NMR (CDCl_3_) δ = 7.79 (dd, 2H), 7.56 (dd, 2H), 3.55 (d, 2H), 1.81 (m, 1H), 1.27 (m, 8H), 0.88 (t, 6H); ^13^C-NMR (CDCl_3_) δ =198.30, 134.91, 133.02, 123.34, 48.02, 38.44, 30.83, 28.75, 24.21, 23.21, 14.25, 11.04; *m*/*z* (ESI) 291.1126 (M^+^); Elemental analysis calculated (%): C, 65.93; H, 7.26; N, 4.81. Found (%): C, 65.15; H, 7.16; N, 4.94.

### 3.4. Optimization of Reaction Conditions for DHAP 

To determine the appropriate conditions for DHAP, a model reaction involving the coupling between PhI and dibromobithiophene was carried out under eight sets of conditions. Because pivalic acid (PivOH) played a key role in DHAP [62,63,64], the reaction conditions for all DHAP involved PivOH as the additive and anhydrous toluene as the solvent. So we choose the different palladium catalysts, bases and phosphine ligands to explore the optimal reaction condition. The results were summarized in Table 2.

The optimized reaction conditions indicated in Table 2 (5 mol % Pd_2_(dba)_3_, 10 mol % P-(o-OMePh)_3_, 2.5 equiv. K_2_CO_3_, 30 mol % PivOH and anhydrous toluene) was subsequently applied to the preparation of a series of PhI-based conjugated polymers starting from the parent PhI and thionated PhI acceptor units with dibromobithiophene donor units, respectively.

#### 3.4.1. DHAP of PhI and Dibromobithiophene (P1)

PhI (0.1297 g, 0.50 mmol), dibromobithiophene (0.3303 g, 0.50 mmol), K_2_CO_3_ (0.1728 g, 1.25 mmol), P-(o-OMePh)_3_ (0.0161 g, 0.05 mmol) and PivOH (0.0153 g, 0.15 mmol) were added into a 25 mL Shrek tube with anhydrous toluene (10 mL). After bubbling with nitrogen for 0.5 h, Pd_2_(dba)_3_ (0.0229 g, 0.025 mmol) was added under the oxygen-free environment, then the reaction mixture was heated to 110 °C and stirred for 72 h under a nitrogen atmosphere. Stirring was stopped and the mixture was cooled down to room temperature, then the reaction mixture was dropped into 200 mL of methanol solution, stirred for 2 h, and let stand for another 2 h before filtered. The precipitate was collected and purified by Soxhlet extraction with methanol, followed by chloroform for 24 h, respectively. The chloroform fraction was concentrated under reduced pressure and then poured into methanol solution. The precipitate was collected and dried in vacuo to yield a yellow green solid (0.2463 g, 64.1%). GPC (DCB at 120 °C): M_n_ = 12.2 kg mol^−1^, Mw = 20.3 kg mol^−1^, and PDI = 1.66. ^1^H-NMR (300 MHz, CDCl_3_): δ = 7.36 (s, 2H), 6.98 (s, 2H), 3.58 (d, 2H), 2.53 (t, 4H), 1.58 (m, 1H), 1.27 (m, 48H), 0.87 (t, 12H).

#### 3.4.2. DHAP of PhIS and Dibromobithiophene (P2)

PhIS (0.1376 g, 0.50 mmol), dibromobithiophene (0.3303 g, 0.50 mmol), K_2_CO_3_ (0.1728 g, 1.25 mmol), P-(o-OMePh)_3_ (0.0161 g, 0.05 mmol) and PivOH (0.0153 g, 0.15 mmol) were added into a 25 mL Shrek tube with anhydrous toluene (10 mL). After nitrogen bubbling for 0.5 h, Pd_2_(dba)_3_ (0.0229 g, 0.025 mmol) was added under the oxygen-free environment, then the reaction mixture was heated to 110 °C and stirred for 72 h under the nitrogen atmosphere. Stirring was stopped and the mixture was cooled down to room temperature, then the reaction mixture was dropped into 200 mL of methanol solution, stirred for 2 h, and let stand for another 2 h before filtered. The precipitate was collected and purified by Soxhlet extraction with methanol, followed by chloroform for 24 h, respectively. The chloroform fraction was concentrated under reduced pressure and then poured into methanol solution. The precipitate was collected and dried in vacuo to yield a reddish brown solid (0.1759 g, 43.3%). GPC (DCB at 120 °C): M_n_ = 9.4 kg mol^−1^, Mw = 26.9 kg mol^−1^, and PDI = 2.87. ^1^H-NMR (300 MHz, CDCl_3_): δ = 7.16 (d, 1H), 6.99 (d, 1H), 6.83 (s, 2H), 3.49 (d, 2H), 2.52 (t, 4H), 1.58 (m, 1H), 1.27 (m, 48H), 0.88 (t, 12H).

#### 3.4.3. DHAP of PhISS and Dibromobithiophene (P3)

PhISS (0.1455 g, 0.50 mmol), dibromobithiophene (0.3303 g, 0.50 mmol), K_2_CO_3_ (0.1728 g, 1.25 mmol), P-(o-OMePh)_3_ (0.0161 g, 0.05 mmol) and PivOH (0.0153 g, 0.15 mmol) were added into a 25 mL Shrek tube with anhydrous toluene (10 mL). After nitrogen bubbling for 0.5 h, Pd_2_(dba)_3_ (0.0229 g, 0.025 mmol) was added under the oxygen-free environment, then the reaction mixture was heated to 110 °C and stirred for 72 h under a nitrogen atmosphere. Stirring was stopped and the mixture was cooled down to room temperature, then the reaction mixture was dropped into 200 mL of methanol solution and stirred for 2 h, and let stand for another 2 h before filtered. The precipitate was collected and purified by Soxhlet extraction with methanol, followed by chloroform for 24 h, respectively. The chloroform fraction was concentrated under reduced pressure and then poured into methanol solution. The precipitate was collected and dried in vacuo to yield a green solid (0.2174 g, 56.2%). GPC (DCB at 120 °C): M_n_ = 11.5 kg mol^−1^, Mw = 23.1 kg mol^−1^, and PDI = 2.01. ^1^H-NMR (300 MHz, CDCl_3_): δ = 7.03 (s, 2H), 6.84 (s, 2H), 3.57 (d, 2H), 2.52 (t, 4H), 1.43 (m, 1H), 1.29 (m, 48H), 0.89 (t, 12H).

## 4. Conclusions

In summary, we have investigated the DHAP with the carbon-hydrogen bond on the benzene ring and the effect of thionation on the optical, electrochemical and thermal properties in solution and thin film for the first time, and show that introducing thionation could lead to a red-shift of the absorption, reduce the LUMO energy level as well as improve the T_d_. DSC characterization demonstrated that liquid crystalline phases appeared in the thionated polymers. Furthermore, when compared to the parent imides, the thionated derivatives possess lower LUMO energy levels and narrower bandgaps that are attractive for potential applications in organic semiconductor devices such as OLEDs, OFETs and OSCs. These results show that thionation can be used to tune the optical, thermal properties of imide-containing polymers and the LUMO energy levels of imide compounds can be simply stabilized by thionation. When combined with the relative ease of the synthetic transformation, these findings suggest that thionation may be a promising method for the design of novel conjugated polymer materials for organic semiconductor devices.

We prove that this work represents a critical step for broadening the application scope of high performance conjugated polymers that can be synthesized by DHAP. Future studies will be concerned with the DHAP of PhI-like monomers to yield the corresponding polymers, and with thionation of polymers containing PhI type chromophores in the backbone. Moreover, the availability of relevant databases about the research on DHAP based on benzene ring and thionation of carbonyl is quite insufficient. Therefore, development of new polymerization processes and thionated molecules is necessary to prepare relevant materials and improve the quantitative understanding of structure–performance relationship, which can potentially optimize the performance of charge mobility as well as power conversion efficiency.

## Figures and Tables

**Table 1:** Optical, thermal and electrochemical properties of P1, P2 and P3.

Polymer	M_n_ (kDa)	PDI	T_d_ (°C)	λ_max_ (nm)	λ_onset_ (nm)	$E_{g}^{opt}$ (eV)	*E_HOMO_* (eV)	*E_LUMO_* (eV)
P1	12.2	1.66	239	322	459	2.70	−5.68	−2.98
P2	9.4	2.87	256	328	468	2.65	−5.93	−3.28
P3	11.5	2.01	298	336	484	2.56	−5.73	−3.17

**Table 2:** Optimization of reaction conditions of PhI and dibromothiophene by DHAP ^a^.

Entry	Pd Catalyst	Base	Ligand	M_n_^b^ (kDa)	PDI ^b^	Yield ^c^ (%)
1	Pd(PPh_3_)_4_	Cs_2_CO_3_	P(o-OMePh)_3_	7.8	3.89	27.6
2	Pd(OAc)_2_	Cs_2_CO_3_	P(o-OMePh)_3_	5.7	3.64	19.7
3	Pd_2_(dba)_3_	Cs_2_CO_3_	P(o-OMePh)_3_	11.0	2.09	49.6
4	Pd(PPh_3_)_2_Cl_2_	Cs_2_CO_3_	P(o-OMePh)_3_	8.9	2.47	22.2
5	Pd_2_(dba)_3_	K_2_CO_3_	P(o-OMePh)_3_	12.2	1.66	64.1
6	Pd_2_(dba)_3_	K_2_CO_3_	P(o-tol)_3_	10.1	1.92	52.4
7	Pd_2_(dba)_3_	K_2_CO_3_	PCy_3_·HBF_4_	8.6	3.49	26.1
8	Pd_2_(dba)_3_	KOAc	P(o-OMePh)_3_	9.1	2.38	39.2

^a^ DHAP was carried out in the presence of palladium catalyst (5 mol %), phosphine ligand (10 mol %), base (2.5 equiv.) and additive (30 mol %) at 110 °C for 72 h. ^b^ Determined by gel permertion chromatography (GPC) on polystyrene standards in dichlorobenzene (DCB). ^c^ The products were obtained by reprecipitation from methanol after Soxhlet extraction.
